# Exposure to the Natural Compound Climacostol Induces Cell Damage and Oxidative Stress in the Fruit Fly *Drosophila melanogaster*

**DOI:** 10.3390/toxics12020102

**Published:** 2024-01-24

**Authors:** Elisabetta Catalani, Kashi Brunetti, Simona Del Quondam, Silvia Bongiorni, Simona Picchietti, Anna Maria Fausto, Gabriele Lupidi, Enrico Marcantoni, Cristiana Perrotta, Gabriele Achille, Federico Buonanno, Claudio Ortenzi, Davide Cervia

**Affiliations:** 1 Department for Innovation in Biological, Agro-Food and Forest Systems (DIBAF), Università degli Studi della Tuscia, 01100 Viterbo, Italy; ecatalani@unitus.it (E.C.); kashi.brunetti@unitus.it (K.B.); simona.delquondam@unitus.it (S.D.Q.); picchietti@unitus.it (S.P.); fausto@unitus.it (A.M.F.); 2 Department of Ecological and Biological Sciences (DEB), Università degli Studi della Tuscia, 01100 Viterbo, Italy; bongiorni@unitus.it; 3 School of Science and Technology, Section of Chemistry, Università degli Studi di Camerino, 62032 Camerino, Italy; gabriele.lupidi@unicam.it (G.L.); enrico.marcantoni@unicam.it (E.M.); 4 Department of Biomedical and Clinical Sciences (DIBIC), Università degli Studi di Milano, 20157 Milano, Italy; cristiana.perrotta@unimi.it; 5 Laboratory of Protistology and Biology Education, Department of Education, Cultural Heritage, and Tourism (ECHT), Università degli Studi di Macerata, 62100 Macerata, Italy; g.achille@unimc.it (G.A.); federico.buonanno@unimc.it (F.B.); claudio.ortenzi@unimc.it (C.O.)

**Keywords:** secondary metabolite, *Drosophila melanogaster*, larvae development, cell damage, redox homeostasis, gastrointestinal tract

## Abstract

The ciliate *Climacostomum virens* produces the metabolite climacostol that displays antimicrobial activity and cytotoxicity on human and rodent tumor cells. Given its potential as a backbone in pharmacological studies, we used the fruit fly *Drosophila melanogaster* to evaluate how the xenobiotic climacostol affects biological systems in vivo at the organismal level. Food administration with climacostol demonstrated its harmful role during larvae developmental stages but not pupation. The midgut of eclosed larvae showed apoptosis and increased generation of reactive oxygen species (ROS), thus demonstrating gastrointestinal toxicity. Climacostol did not affect enteroendocrine cell proliferation, suggesting moderate damage that does not initiate the repairing program. The fact that climacostol increased brain ROS and inhibited the proliferation of neural cells revealed a systemic (neurotoxic) role of this harmful substance. In this line, we found lower expression of relevant antioxidant enzymes in the larvae and impaired mitochondrial activity. Adult offsprings presented no major alterations in survival and mobility, as well the absence of abnormal phenotypes. However, mitochondrial activity and oviposition behavior was somewhat affected, indicating the chronic toxicity of climacostol, which continues moderately until adult stages. These results revealed for the first time the detrimental role of ingested climacostol in a non-target multicellular organism.

## 1. Introduction

Climacostol (5-[(2*Z*)-non-2-en-1-yl]benzene-1,3-diol) is a secondary metabolite produced by the free-living ciliated protist *Climacostomum virens* with a dual natural role, defense against unicellular and multicellular predators and offense to promote its feeding, mediating predator–prey interactions [1,2]. The toxin is stored in peculiar ejectable organelles of the protozoan called extrusomes [3], and when discharged causes severe necrotic damage and total lysis of unicellular predators in a matter of seconds [4]. In addition to its physiological activity, the toxin exerts antimicrobial activity against pathogenic Gram-positive bacteria and the fungus *Candida albicans*, whereas it is ineffective against Gram-negative strains [5]. Furthermore, preliminary assessment of climacostol’s role against human adenovirus 5 (HAdV5) revealed its ability to reduce HAdV5 infectivity in vitro [6].

Most importantly, climacostol displays cytotoxic activity on some human and rodent tumor cells lines, activating a mitochondrion-dependent apoptotic program and inhibiting cell growth [7,8,9]. It was also observed that climacostol can induce the generation of reactive oxygen species (ROS) and the cleavage of nuclear DNA, with cellular damage together with mitochondrial dysfunction, which contributes to cancerous selective cell death [7,8,10,11].

In vivo mice studies have shown that climacostol inhibits the growth of melanoma allografts after intratumoral injections, reduces the viability and the proliferation of tumor cells, and decreases the microvessel sprouting inside the tumor [7]. A significant increment in the survival of transplanted mice was observed, as well as a decrease in tumor weight and size. Further analyses revealed that climacostol, as well as apoptosis induction, selectively impairs autophagy [8] probably acting separately on the two pathways that are pivotal in the life/death decisions of the cell [12].

Due to its noteworthy characteristics, new analogs and chemically modified climacostol molecules were further investigated [4,13]. These organic synthesis-obtained compounds display interesting additional properties, including prodrug potential, and most importantly, offer the chance to investigate the application of a cytotoxic compound by circumventing bioavailability issues. In particular, the analog AN1 (2-methyl-5 [(2Z)-non-2-en-1-yl]benzene-1,3-diol) exhibits higher toxicity than climacostol against pathogen microbes and protists; the analog AN2 (5-[(2Z)-non-2-en-1-yl]benzene-1,2,3-triol) is able to induce apoptosis in free living protists. A third synthetic analog, MOMO, is stable and non-toxic under physiological conditions (neutral pH) but can be efficiently triggered to restore the cytotoxic activity of climacostol in mild acidic environments. However, despite the potential of native climacostol as a backbone in pharmacological studies, no data are available on how it affects biological systems at the organismal level.

In parallel with traditional vertebrate systems, the use of the fruit fly *Drosophila melanogaster* for exploring pathophysiological alterations increased in the last years together with its potential use in drug discovery, including the research on bioactive natural compounds [14,15,16,17,18,19]. Biological settings are very well conserved in Drosophila and homologs of ca. 75% of human pathogenic genes have been established. Also, in the context of a well-known and powerful genetic framework, Drosophila is beneficial for animal husbandry and has a short generation time and lifespan. For these reasons, *D. melanogaster* is as a valuable model to understand how a xenobiotic perturbs a biological multicellular system, also concerning biochemical and molecular characteristics [18,20,21,22,23,24].

In agreement with previous studies using natural compounds [25,26], flies were used here as a rapid and low-cost model to assess the accessibility and detrimental activity of native climacostol when supplemented in the diet, since the feeding experiments are important as a first step towards investigating the biological effects of molecules in vivo [27,28,29]. In particular, we studied the damages induced by oral-delivered native climacostol on *D. melanogaster* at mating/developmental levels. Its effects on the homeostasis of the gastrointestinal tract, which is primarily exposed to the toxin, and brain (neurotoxicity) upon absorption were also evaluated, including apoptotic events and oxidative stress-related signals.

In normal settings, humans are not exposed to the climacostol toxin. However, its potential in drug research prompted us to investigate the systemic effects of the toxin in vivo. Our results revealed for the first time the detrimental role of ingested climacostol in a non-target multicellular organism.

## 2. Materials and Methods

### 2.1. Chemicals

Chemically synthesized climacostol (C_15_H_22_O_2_, 5-[(2*Z*)-non-2-en-1-yl]benzene-1,3-diol) [7,30] was dissolved in absolute ethanol (stock solution: 100 mg/mL) and stored in the dark at −20 °C until use. Bovine serum albumin (BSA), normal goat serum, anti-nitrotyrosine primary antibody (#A21285), and Alexa Fluor secondary antibody (#A21428) were purchased from Thermo Fisher Scientific (Monza, Italy). Anti-cleaved caspase 3 primary antibody (#9664) was purchased from Cell Signaling Technology (Danvers, MA, USA), while anti-prospero (MR1A) primary antibody was obtained from the Developmental Studies Hybridoma Bank (University of Iowa, Iowa City, IA, USA). The primer pairs for PCR analysis were purchased from Bio-Fab Research (Rome, Italy). In cases where they are not indicated, the other reagents were purchased from Merck Sigma-Aldrich (Darmstadt, Germany).

### 2.2. Fly Husbandry and Treatments

All experiments were performed with female and male adult *D. melanogaster* (Oregon-R strain from Bloomington Drosophila Stock Center, Indiana University Bloomington, IN, USA). Flies were routinely raised in an incubator at 25 ± 1 °C and 12 h/12 h light/dark cycles (light-on time set at 6 am every day) on standard corn meal agar food. Fly food was prepared as follows: 25 g of yellow cornmeal, 25 g of brewer’s yeast, 2 g of agar, and 27 g of sucrose (9% *w*/*v*) were mixed and dissolved by adding warm plain water to a final volume of 300 mL, the hydration source of the flies. The mixture was autoclaved and the broad-spectrum fungicide Nipagin (0.75 g) was added at ca. 50 °C. Subsequently, 10 mL of cooling down mixture was dispensed into vials to reach ca. 25 °C. Flies were cultured for one generation at constant density prior to exposure (mating flies or eggs) on diets supplemented with climacostol (experimental diet). In agreement with previous data using similar compounds [13], climacostol was added to the food at a concentration ranging from 100 to 300 µg/mL. In particular, climacostol was mixed to cooling down food into vials to obtain the final working concentration, making sure the concentration of ethanol (vehicle) was <0.5% to avoid unrelated effects. Although much flexibility in terms of the carrier solvent is allowed in the preparation of drug-infused-fly-food [31], our choice for ethanol vehicle comes from the fact that synthetic climacostol dissolved in absolute ethanol is highly stable in the dark at −20 °C [7,30]. A standard diet with the climacostol vehicle was used as the control diet.

### 2.3. Mating Procedure and Developmental Assays

Populations of adult flies (3 days old) were placed in vials (15 females and 10 males) for mating and egg laying. Vials were visually inspected to ensure copulation was occurring; within 5–30 min, all females were typically paired with males. After 3 days, the flies were removed, and larval development was checked twice daily. In particular, the number of third instar larvae emerged from food (control and climacostol-supplemented diet) was recorded. Individual eggs were also gently picked after 24 h of copulation of untreated adults using 2% agar plates supplemented with apple juice. We added phosphate-buffered saline (PBS) onto the plate and gently wiped the apple-agar surface with a soft-thin brush. Eggs were then washed in PBS, counted and separated under a stereo microscope in new food (control and climacostol-supplemented diet) vials (100 eggs each), before counting twice daily the third instar larvae and eclosed adults. In other words, climacostol exposure started from fertilized eggs to larvae/adult eclosion. When indicated, Drosophila females of the first offspring generation (F1) eclosed from both control and climacostol-treated eggs were collected as virgins, fed with the standard diet and then housed with males. The individual laid eggs after mating were counted on day 3. For size measurements, third instar larvae emerged from food were considered. Larval length was measured by graph paper. To determine weight, larvae were frozen and weighed using an ultramicro balance (with high resolution of up to 0.0001 mg).

### 2.4. Fluorescence Confocal Microscopy

Third instar larvae were immersion-fixed overnight in 4% paraformaldehyde in 0.1 M PB at 4 °C, transferred to 25% sucrose in PB, and stored at 4 °C for at least 48 h. Sections (16 µm) were obtained by a cryostat, mounted onto positive charged slides and stored at −20 °C until use. To allow proper comparison in midgut (i.e., A7 abdominal segment) and brain analysis, the same depth/region of the structure was sectioned. The TUNEL method (DeadEnd Fluorometric TUNEL System, Promega Corporation, Milano, Italy) was performed according to the manufacturer’s instructions and Mounting Medium with 4′,6-diamidine-2′-phenylindole dihydrochloride (DAPI) (#ab104139, Abcam, Cambridge, UK) was used for nuclei detection. For immunostaining, sections were washed in PB and then pre-incubated for 30 min at room temperature with 5% BSA and 10% of normal goat serum in PB containing 0.5% Triton X-100. Pre-treated sections were incubated overnight at 4 °C with the rabbit primary antibodies anti-cleaved caspase 3 (1:500) and anti-nitrotyrosine (1:100), and the mouse primary antibody anti-prospero (1:30) in PB containing 0.5% Triton X-100. Following washes in PB, the sections were incubated in the Alexa Fluor goat anti-rabbit 555 secondary antibody (1:200) in PB overnight at room temperature. When appropriate, the slides were coverslipped with Mounting Medium with DAPI for nuclei detection. Incubation in secondary antibodies alone was routinely performed as a negative control. Images were acquired by a Zeiss LSM 710 confocal microscope (Carl Zeiss, Oberkochen, Germany) and the distance between adjacent focal planes (z-stacks) was set at 1 µm.

### 2.5. Mitotic Index

To analyze mitotic parameters, brains from the third instar larvae were dissected in hypotonic solution (0.8% sodium citrate) and fixed at room temperature in a freshly prepared mixture of acetic acid/methanol/H_2_O (11:12:2) for 30 s. Single fixed brains were then transferred into small drops of 45% acetic acid on a very clean, dust-free non-siliconized coverslip for 2 min. A clean slide was lowered onto the coverslip and squashed. Slide was frozen in liquid nitrogen, the coverslip was removed with a razor blade and the slide was immediately immersed in absolute ethanol at −20 °C for 15 min. Slides were air-dried and DAPI stained, with 0.2 µg/mL in 2× saline sodium citrate (20×: 0.15 M NaCl, 0.015 M sodium citrate). The mitotic index was defined as the number of mitotic cells per optical field. The optical field was the circular area defined by a 100× Zeiss objective/1.30 Plan-NEOFLUAR, using 10× oculars and the Optovar set at 1.25 (Zeiss Axiophot microscope). Every optical field occupied by brain tissue was scored (at least 50–100 optical fields per slide).

### 2.6. RNA Extraction and PCR

The analysis of mRNA expression in ca. 5 third instar larvae was performed in PureZOL reagent (Bio-Rad, Hercules, CA, USA). Total RNA (500–800 µg) was retro-transcribed using the iScript gDNA Clear cDNA Synthesis Kit (Bio-Rad). Quantitative PCR (qPCR) was performed using SsoAdvanced™ Universal SYBR Green Supermix (Bio-Rad) and the CFX96 Touch Real-Time PCR Detection System (Bio-Rad). The primer pairs are detailed in Table 1. Rpl32 has been used as a housekeeping gene for normalization by using the 2^−ΔΔCT^ method [32].

### 2.7. 3-(4,5-Dimethylthiazol-2-yl)-2,5-diphenyltetrazolium Bromide (MTT) Assay

The mitochondrial viability in whole body homogenates of third instar larvae and adults was tested as previously published [25,33]. Briefly, 20 larvae or adults were weighed and manually homogenized in cold PB. The supernatants were collected after each consecutive centrifugation at 4 °C, for 5 min, at 1500 and 1000 rpm, respectively. Mitochondrial activity was then evaluated using the MTT reduction method (0.5 mg/mL of final concentration) at an absorbance of 595 nm.

### 2.8. Survival and Climbing

Adult fly survivorship was documented recording the numbers of dead individuals per vial every 3 days, starting at the day of adult emergence. Geotaxis was assessed at day 20 from eclosion using a climbing assay (negative geotaxis reflex in opposition to the Earth’s gravity) [26]. *D. melanogaster* flies were separated into cohorts (empty vials) consisting of ca. 20 flies. A horizontal line was drawn 15 cm above the bottom of the vial. After a 10 min rest period, the flies were tapped to the bottom of the vials before starting to climb (vertical walking). The number of flies that climbed up to the 15 cm mark after 60 s was recorded as the percentage success rate. A camera was recording fly movement during the experiment. Each trial was performed three times, at 1 min intervals, and the results were averaged.

### 2.9. Scanning Electron Microscopy (SEM)

*D. melanogaster* flies were fixed and dehydrated as previously described [26]. Samples were dried by the critical point method using CO_2_ in a Balzers Union CPD 020 apparatus (Balzers Union Limited, Balzers, Liechtenstein). Then, the samples were attached to aluminum stubs using carbon tape and sputter-coated with gold in a Balzers Union MED 010 unit. The observations of eyes and wings were made by a JSM 6010LA electron microscope (Jeol, Tokyo, Japan).

### 2.10. Statistical Analysis

Generally, sample size calculation was conceptualized with 5% alpha error, 80% power and appropriate effect strength. Samples were only excluded from analyses due to technical problems, e.g., pipetting error, loss/spill of samples, or defects in materials/hardware. The F-test was performed to evaluate the homogeneity of variance and Shapiro–Wilk test was used for evaluating data normality. The statistical significance of raw data between the groups (completely randomized) in each experiment was evaluated using unpaired Student’s t (single comparisons) or one-way ANOVA followed by the Tukey post-test (multiple comparisons). A *p*-value ≤ 0.05 was considered statistically significant. Data belonging to different experiments were represented and averaged in the same graph. The GraphPad Prism 6 software package (GraphPad Software, San Diego, CA, USA) was used. The results were expressed as means ± SEM of the indicated *n* values.

## 3. Results

### 3.1. Effects on Development

**Figure 1:**
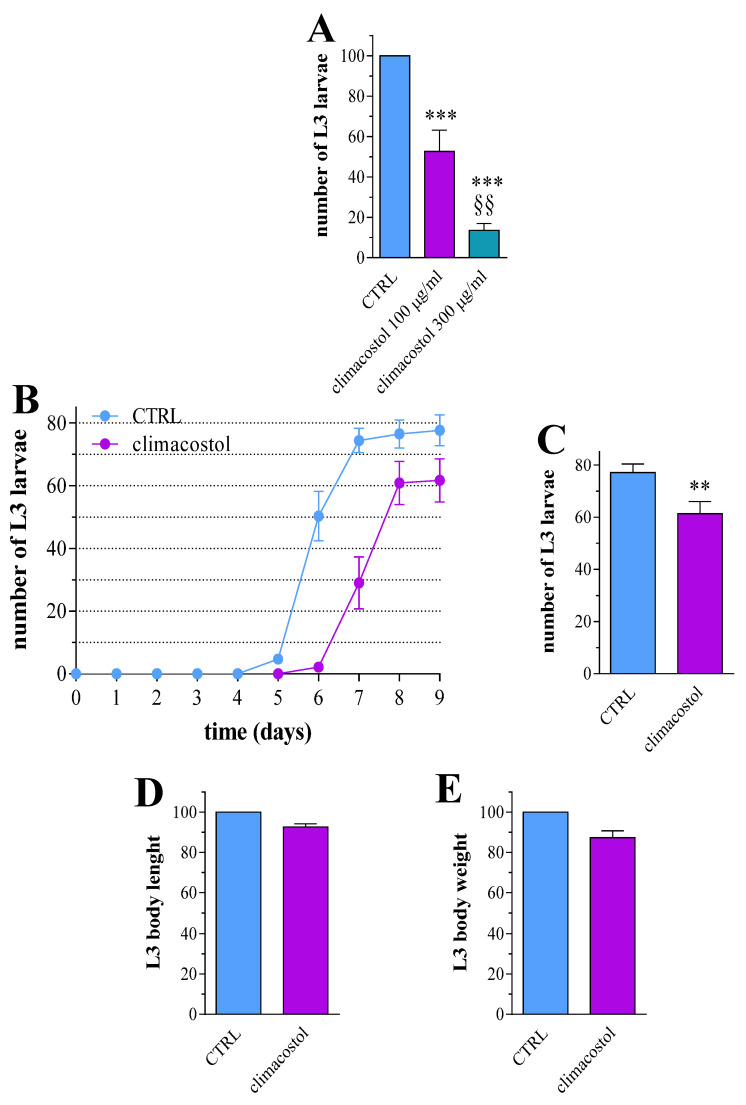
Climacostol impairs Drosophila development. (**A**) The number of third instar larvae developed from mating adults both in the absence (control, CTRL) and in the presence of increasing concentrations of climacostol in the food. Results are expressed as the percentage of CTRL larvae. *** *p* < 0.0001 vs. CTRL; §§ *p* < 0.001 vs. 100 µg/mL climacostol. Data have been obtained from 5 independent experiments. (**B**) Time from fertilized eggs (100) to third instar larvae eclosion represented by cumulative number of larvae emerging from CTRL food and food supplemented with 100 µg/mL climacostol. (**C**) Number of eclosed third instar larvae on day 8–9 of egg treatment. ** *p* < 0.001 vs. CTRL. Data have been obtained from 8 independent experiments. (**D**) Body length and (**E**) body weight of eclosed third instar larvae. Results are expressed as the percentage of body traits in CTRL group. Data have been obtained from 8 independent experiments (50 animals).

*D. melanogaster* exhibits a four-step life cycle: egg, larva, pupa, and adult fly [34]. Eggs are deposited on the food, and in less than 24 h, the first instar larvae hatch from eggs and eat continuously, except for moulting after first instar and second instar stages. Only at the end of third instar step, larvae exit from the food for pupariation and metamorphosis, and the adult flies eclose. Equal numbers of mating flies were placed in vials containing a standard Drosophila diet (control diet) and mixed with climacostol (experimental diet) at different concentrations. Adult flies were removed after egg laying, and larval survival was measured as the number of third instar larvae (L3) leaving the food. When compared to the control, third instar larvae were significantly reduced in both 100 and 300 µg/mL climacostol-treated groups (Figure 1A). The results showed a dose-dependent response to treatments. In particular, a drastic toxic effect on mating/eclosion was achieved with 300 µg/mL climacostol since very few larvae emerged from the food while 100 µg/mL had intermediate effects. This appeared to be the best working dose to be further tested in our system. To better determine if in ovo treatment with 100 μg/mL climacostol affected Drosophila development, an equivalent number of fertilized eggs were placed in vials containing the control and experimental diet. As shown in Figure 1B, climacostol intake clearly retarded first pupation by delaying the time to the third instar larvae appearance and also affected the total number of emerging L3. In particular, developed L3 were significantly decreased by ca. 20% (Figure 1C). Then, third instar larvae were collected and analyzed for their body size, as a convenient indicator of larvae growth. Larvae that emerged from climacostol-supplemented food had comparable body traits, i.e., body length and weight, to the control-fed group (Figure 1D,E).

### 3.2. Cell Damage

**Figure 2:**
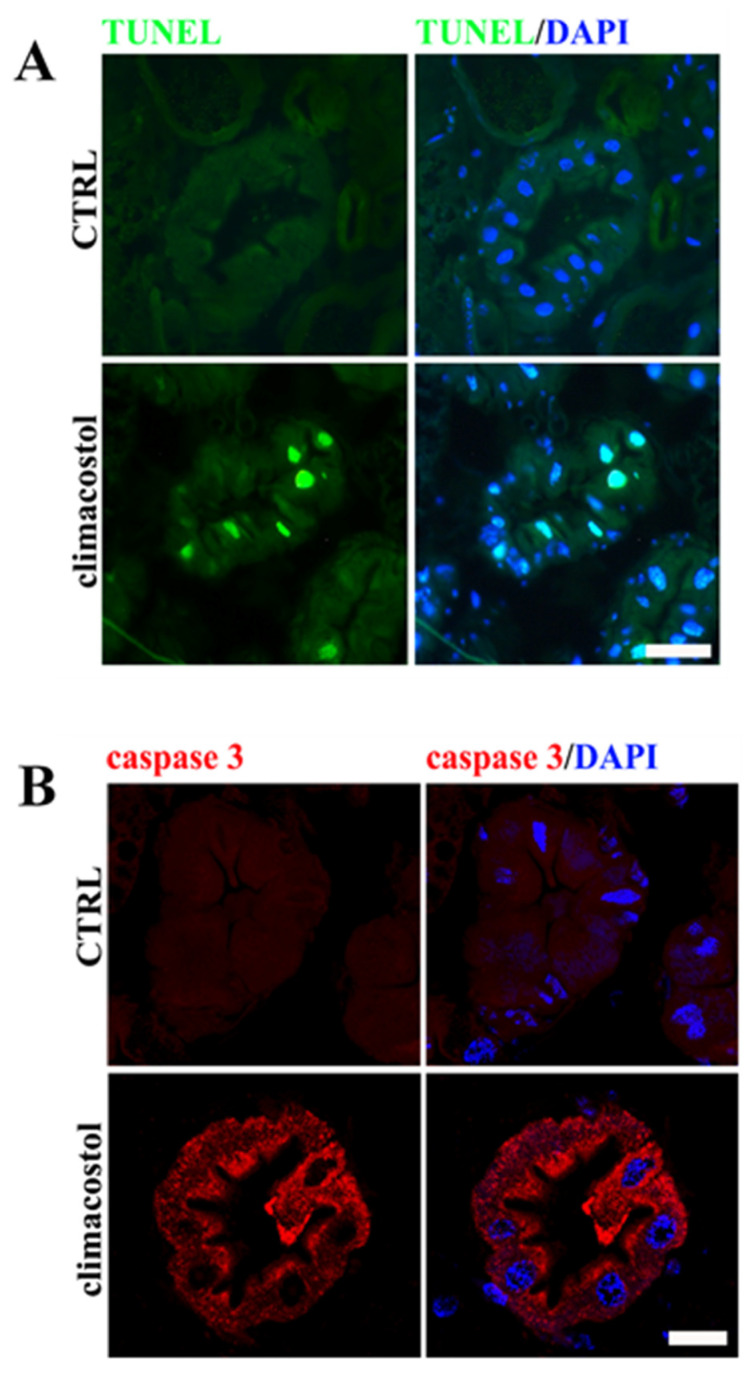
Climacostol-induced damage in Drosophila midgut. Confocal microscopy imaging of TUNEL labeling (**A**) and cleaved caspase 3 immunostaining (**B**) in midgut sections of third instar larvae eclosed both in the absence (control, CTRL) and in the presence of 100 µg/mL climacostol. DAPI was used for nuclei detection. Scale bars: 50 μm. Images are representative of 20 animals obtained from 4 independent experiments.

The oral toxicity of 100 μg/mL climacostol was then investigated in third instar larvae by first assessing the typical features of apoptotic cell death in the midgut, which corresponds to the small intestine of humans and is optimally placed to come into direct contact with ingested drugs [35]. In recent years, the Drosophila midgut digestive tract has been used as an excellent system to study the homeostasis of intestinal epithelium [14,36,37,38,39]. As shown in Figure 2A, the digestive tract of larvae that emerged from climacostol-supplemented food showed apoptotic DNA fragmentation. Indeed, TUNEL^+^ nuclei were clearly detected in midgut cells. Accordingly, cleaved (active) caspase 3 staining was found in the cytoplasm of midgut cells in the same cell death area (Figure 2B).

**Figure 3:**
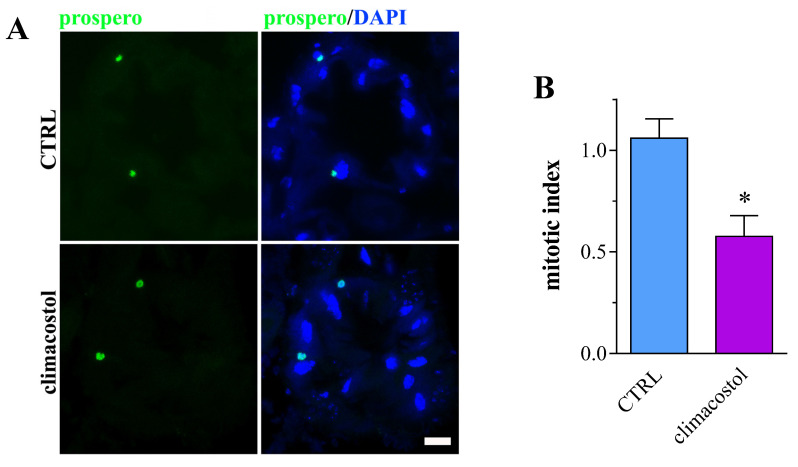
(**A**). Confocal microscopy imaging of prospero immunostaining in midgut sections of third instar larvae eclosed both in the absence (control, CTRL) and in the presence of 100 µg/mL climacostol. DAPI was used for nuclei detection. Scale bar: 20 μm. Images are representative of 15 animals obtained from 3 independent experiments. (**B**) Mitotic index analysis in brains from the third instar larvae. Results are expressed as fold change in CTRL. * *p* < 0.01 vs. CTRL. Data have been obtained from 4 independent experiments (15 animals).

Third instar larvae treated with 100 μg/mL climacostol and control samples express similar immunostaining patterns of the secretory enteroendocrine cell marker prospero [40,41] in the midgut (Figure 3A). However, harmful systemic effects were detected when brains were used for the analysis of the mitotic cell cycle. As shown in Figure 3B, the larvae fed with climacostol had a significantly reduced mitotic index (ca. 45% decrease) versus larvae fed with the standard diet, consistent with a lower proliferation rate of neuroblasts and neurotoxicity.

### 3.3. Redox State

**Figure 4:**
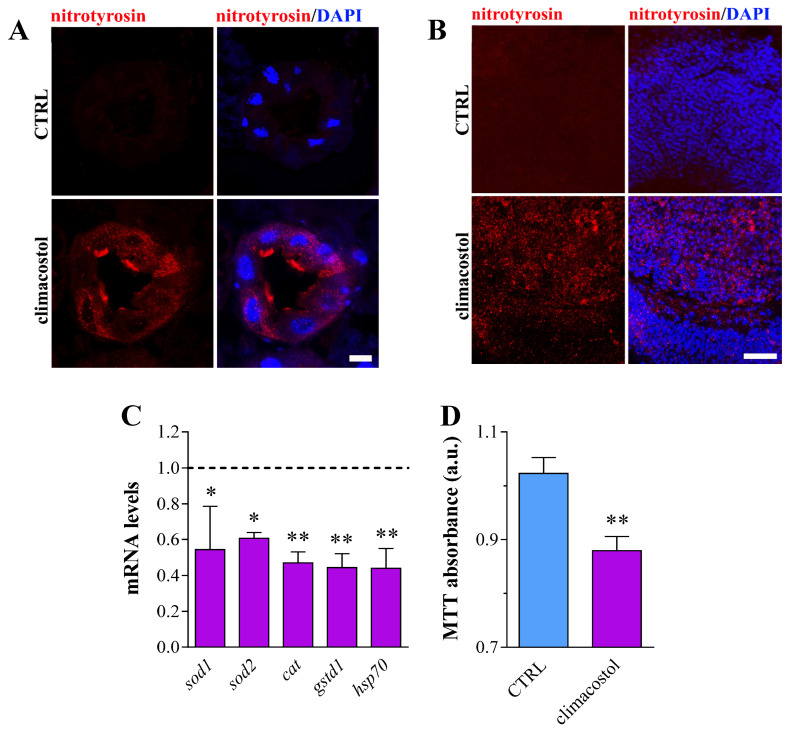
Climacostol affects redox homeostasis of Drosophila. Confocal microscopy imaging of nitrotyrosine immunostaining in midgut (**A**) and brain (**B**) sections of third instar larvae eclosed both in the absence (control, CTRL) and in the presence of 100 µg/mL climacostol. DAPI was used for nuclei detection. Scale bars: 20 μm. Images are representative of 15 animals obtained from 4 independent experiments. (**C**) mRNA levels of sod1, sod2, cat, gstd1, and hsp70 genes by qPCR in third instar larvae eclosed in the presence of 100 µg/mL climacostol. Results are expressed as fold change in CTRL (dotted line). Data have been obtained from 4 independent experiments (20 animals). (**D**) Measurement of mitochondrial activity by MTT absorbance in third instar larvae. Results are expressed as arbitrary units (a.u.). Data have been obtained from 3 independent experiments (60 animals). * *p* < 0.01 and ** *p* < 0.001 vs. CTRL.

Next, we determined the oxidative stress in the digestive tract of third instar larvae exposed to 100 μg/mL climacostol. The increase in ROS generation was demonstrated by fluorescence immunostaining to detect peroxynitrite levels using an anti-nitrotyrosine antibody. Indeed, midgut cells under climacostol exhibited higher peroxynitrite labeling than the control group (Figure 4A). Similar results were obtained in brain slices (Figure 4B). The alteration of redox homeostasis was confirmed by qPCR analysis of conserved prototypical oxidative stress response genes [42,43]. As shown in Figure 4C, the mRNA expression of superoxide dismutase (SOD) 1 and 2, catalase (CAT), and glutathione-S-transferase (GstD1) significantly decreased in whole-body larvae treated with climacostol. We also found a marked inhibition of Heat Shock Protein 70 (HSP70) mRNA levels, the most important inducible heat shock protein in flies [44,45]. Along this line, these animals also displayed impaired mitochondrial activity. Indeed, we found a significant decrease in MTT reductive ability in larvae fed with climacostol when compared with the control condition (Figure 4D).

### 3.4. Long-Term Toxicity

**Figure 5:**
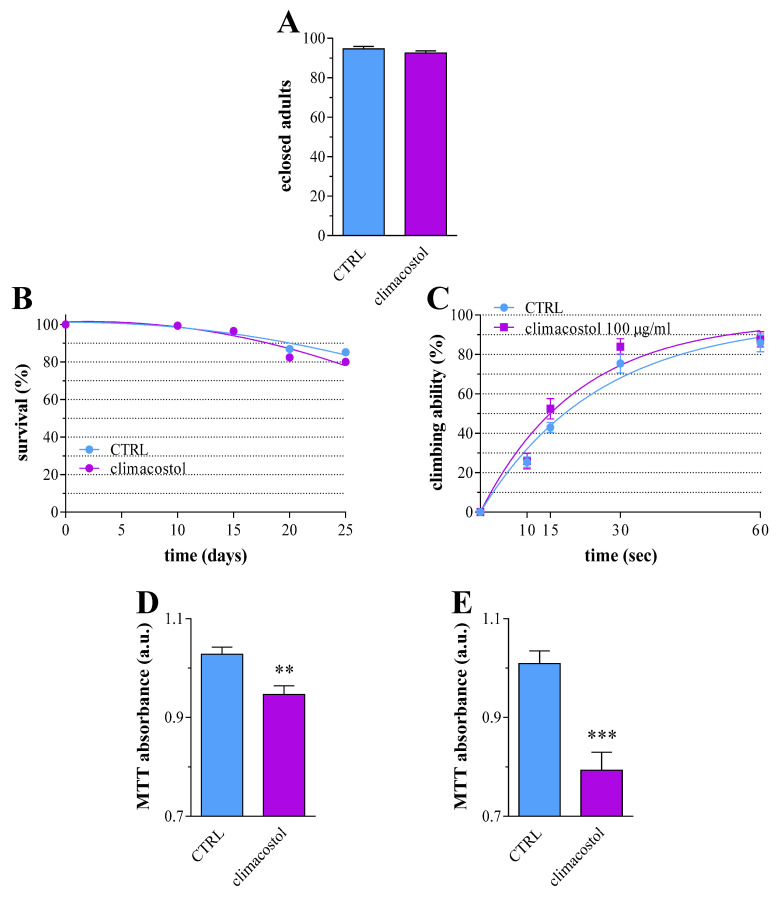
Long-term effects of climacostol in Drosophila. (**A**) Number of adults eclosed from third instar larvae developed in the absence (control, CTRL) and in the presence of 100 µg/mL climacostol in the food. Results are expressed as percentage of pupae. Data have been obtained from 3 independent experiments. Eclosed adults were then fed with a standard diet. (**B**) Survival of adults, evaluated by the total number of living flies during the experimental period. Results are expressed as a percentage of eclosed adults at day 0. Data have been obtained from 3 independent experiments (90 animals). (**C**) Climbing ability of adults on day 20 after eclosion. The number of flies that climbed up to the 15 cm mark at 10, 15, 30, and 60 sec was recorded and expressed as the percentage of success rate. Data have been obtained from 3 independent experiments (60 animals). Measurement of mitochondrial activity by MTT absorbance in adults at day 10 (**D**) and 20 (**E**) from eclosion. Results are expressed as arbitrary units (a.u.). Data have been obtained from 3 independent experiments (60 animals). ** *p* < 0.001 and *** *p* < 0.0001 vs. CTRL.

We then investigated whether the detrimental effects of climacostol after in ovo administration were limited to the larval stages. To this end, the percentage of developed adults, i.e., the amount of adults out of the total number of L3, was comparable in 100 μg/mL climacostol-treated and control samples (Figure 5A), thus indicating a detrimental effect at larval stages not affecting pupation viability. In another set of experiments, adults eclosed from both the control and 100 µg/mL climacostol-supplemented food were fed with a standard diet. First, their general behavior and their survival rate were evaluated. Climacostol did not affect the survival of adult Drosophila throughout the experimental timeline (Figure 5B). Accordingly, no damaging effects were detected at visual observations. We then analyzed the mobility of adult flies by measuring their climbing capability at day 20 from eclosion. As shown in Figure 5C, the climbing ability of the climacostol-treated group was comparable with the control. On the other hand, a significant decrease in MTT reductive ability was achieved in 10- and 20-day-old flies (Figure 5D,E), suggesting a somewhat detrimental chronic condition.

**Figure 6:**
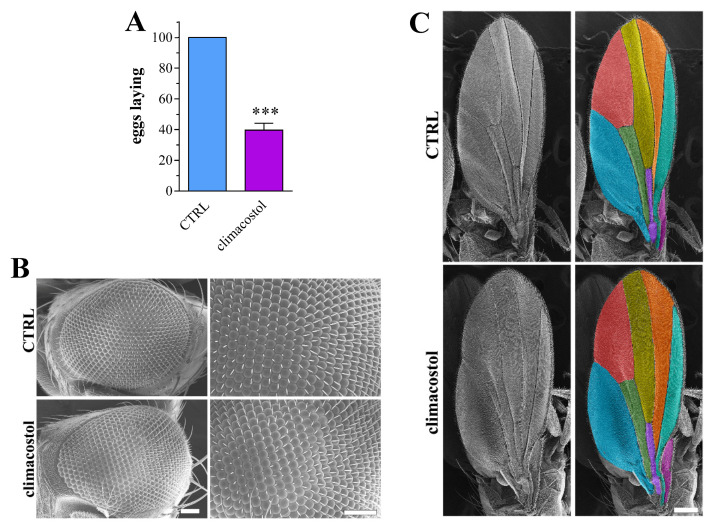
Long-term effects of climacostol in Drosophila. Adults eclosed from third instar larvae developed in the absence (control, CTRL) and in the presence of 100 µg/mL climacostol in the food were then fed with a standard diet. (**A**) Number of laid eggs when adult females were collected as virgins before copulation. Results are expressed as percentage of CTRL. Data have been obtained from 3 independent experiments. *** *p* < 0.0001 vs. CTRL. Adult (10 days-old) Drosophila of the F2 generation were analysed by SEM. (**B**) Eye phenotype. Right panels depict magnificated images showing properly arranged ommatidia and bristles. Scale bars: 50 μm. (**C**) Wing phenotype. Different regions of wings are color labelled in the right panels. Scale bar: 200 μm. Images are representative of 20 animals obtained from 4 independent experiments.

In this respect, climacostol diets were identified as affecting Drosophila oviposition. Indeed, when F1 offspring adult females (eclosed from both control and climacostol-treated eggs and then fed a standard diet) were collected as virgins before copulation, the number of laid eggs in the climacostol group was significantly lower than the control (Figure 6A). However, the climacostol-fed larvae upon reaching adulthood (both F1 and F2 generations) did not develop structural abnormalities. Indeed, as revealed by SEM studies, the compound eyes and wings of the F1 and F2 adults maintained a unique and stable pattern without alterations in morphology and symmetry (Figure 6B,C).

## 4. Discussion

Natural products exhibit an enormous range of structural and chemical diversity, and in particular, small organic molecules derived from nature demonstrate significant translational potential [46,47]. This is ascribed to the remarkable ability of living organisms to synthesize elaborate molecules with well-defined properties in biological systems, and climacostol serving as a demonstrative example of a small molecule. The direct purification of natural climacostol from cell cultures is severely problematic, yielding only minimal amounts of toxin [4]. Consequently, many efforts have been undertaken to address this limit, focusing on the development of efficient strategies for synthesizing appreciable amounts [30]. These efforts aim to achieve cost-effective and expedited production processes and this allowed us to carry out studies on multicellular and complex organisms such as Drosophila. Indeed, Drosophila is particularly useful for phenotype-based approaches to xenobiotics as it provides an opportunity to test compounds in a whole animal setting where efficacy and systemic toxicity can be monitored simultaneously [21,48,49,50]. In this respect, small molecules are able to interact with different biological targets and this may lead to damaging events, which are important from a pharmacological point of view [51,52,53,54,55]. Here, we evaluated the effects of native climacostol on Drosophila homeostasis.

Our feeding experiments demonstrated the oral bioavailability and activity of climacostol in vivo as the first step towards deciphering its biological effects in a non-target multicellular organism. Oral bioavailability is a convenient aspect of most small molecule drugs [56]. In Drosophila, food passes through the foregut and the midgut where absorption takes place in a manner similar to high vertebrates [35]. The use of a sub-lethal dose in ovo of climacostol clearly demonstrated its harmful role during larvae developmental stages. Of interest, the fact that third instar larvae emerging from both climacostol and control food had comparable body traits indicated no major effects in diet consumption. This is an important aspect to be considered since small differences in nutrient assumption can profoundly affect the health and survival of Drosophila [57,58].

In the midgut of third instar larvae administered with climacostol, we found apoptotic cells and increased generation of ROS, thus demonstrating its gastrointestinal toxicity. ROS is an important factor for gut homeostasis [59] and excessive ROS may be destructive to the intestine epithelial cells of flies [60,61,62]. The Drosophila midgut is a epithelial monolayer with a rapid turnover rate [63]. Chemical insults may damage the epithelium, which, in turn, releases proliferative signals as a local early response [14]. Although ROS are critical messengers for the differentiation and cell growth of fly intestinal stem cells [64,65], in our system, climacostol did not affect the proliferation of secretory enteroendocrine cells, suggesting moderate damage/ROS increase that did not initiate the repairing program. Apoptosis, impaired ROS production, and decreased antioxidant enzymes have been described in the intestine of Drosophila treated with different toxins [60,61,62]. In addition, preliminary observations in flies revealed cytotoxic effects of the climacostol synthetic analog MOMO [13]. The fact that native climacostol increased the production of brain ROS and decreased the proliferation of brain cells suggested a systemic role of this harmful substance, likely able to penetrate the fly gut barrier and affect neuronal homeostasis. In this line, we found that climacostol inhibited the cellular antioxidant enzyme expression, namely SODs, CAT, and GstD1 [43,66], in the larvae. The levels of HSP70, which may act as molecular chaperones counteracting oxidative stress damage [67,68,69], were also decreased. Organismal responses to redox imbalance and unfolded protein response signals involve mitochondria damage [70], which are common between Drosophila and humans [71]. Accordingly, climacostol impaired larvae mitochondrial activity.

Further investigations found that in ovo treatment with climacostol does not affect pupation viability. Indeed, after encapsulation of the third instar larvae, pupae normally developed into adults. Also, eclosed adults did not present major alterations, including survival and mobility. However, mitochondrial activity and oviposition behavior was somewhat inhibited, indicating a chronic detrimental role. Of interest, the adults emerging from both the F1 and F2 offspring generations of climacostol-treated groups did not develop structural abnormalities, therefore suggesting the absence of abnormal phenotype in the progeny. Taken together, these results indicated that the diet administration with climacostol negatively affected larvae development of *D. melanogaster*, inducing apoptosis and high levels of oxidative stress, which might have an important role in intestinal and systemic (neuro)toxicology. Detrimental effects continued moderately until the adult stages, thus showing somewhat chronic damage without involving genotoxic effects. This evidence must be taken into account in the potential use of climacostol as a lead compound.

From an ecological perspective, the possibility of modulating the effects of climacostol molecules on different targets, from viruses to uni- and multicellular organisms, combined with the fact that climacostol is naturally produced by the freshwater ciliate along with a small proportion of related congeners (5-(Z,Z)-undeca-2,5-dienyl-benzene-1,3-diol), and (5-(Z,Z,Z)-undeca-2,5,8-trienyl-benzene-1,3-diol) [2], could be of great environmental utility, including the possible applications in wastewater treatment plants. As recently speculated [6], the incorporation of ciliated protists producing these secondary metabolites into activated sludge in wastewater treatment plants could prove advantageous by reducing the presence of some prokaryotic and eukaryotic pathogens and mitigating the viral load in the secondary effluent. Additionally, attention is drawn to its prospective use in human and animal settings as a chemically obtained prodrug MOMO, selectively activated under acidic conditions [13]. MOMO may counteract some parasitic protozoa belonging to the family Trypanosomatidae, whose intracellular forms (amastigotes) grow and multiply within mammalian host cells where they are sheltered within a strongly acidic parasitophorous vacuole. The peculiar properties of MOMO open the way for targeted applications against host–parasite interaction of Leishmania and Trypanosoma infection. Other evidence suggests climacostol as a highly active compound to be considered for the design of new drugs with cytotoxic activities against cancer, for instance melanoma [4,7,8].

## 5. Conclusions

Comprehensive investigations, such as this one conducted in Drosophila, have become crucial for understanding the effects of native climacostol on animals, including humans. Indeed, it is imperative to emphasize that studies of this nature necessitate preliminary exploration before advancing to pharmacological implementations. However, insects are functionally and evolutionarily far from vertebrates. This means that before translating them to human pathophysiology, fly studies have to be carefully considered. In this respect, the data obtained from Drosophila need to be compared with data from other organisms of increasing biological complexity. Nevertheless, Drosophila is a useful experimental system to be compared with more traditional in vivo models. In this respect, there is a general agreement that Drosophila has a key role in drug discovery, favoring the translation of lead compounds into clinical therapeutics [14,15,72,73,74]. Overall, the data collected in this paper on the systemic toxicity of climacostol represent a starting point to plan possible alternative treatments of infective or/and neoplastic diseases, such as cancer or protozoan parasitosis, by modified non-toxic and effective analogs of the title compound.

## Figures and Tables

**Table 1:** Primer pairs designed for qPCR analysis.

Gene Name	FlyBase ID	Primer Sequence	Amplicon Size
*sod1*	FBgn0003462	F: 5’-ACCGACTCCAAGATTACGCTC-3’ R: 5’-CAGTGGCCGACATCGGAATA-3’	197 bp
*sod2*	FBgn0010213	F: 5’-AATCTAAATGCCGCCGAGGA-3’ R: 5’-CTCTTCCACTGCGACTCGAT-3’	197 bp
*cat*	FBgn0000261	F: 5’-CTATGGCTCGCACACCTTCA-3’ R: 5’-TCGTCCAACTGGGGAACTTG-3’	194 bp
*gstd1*	FBgn0001149	F: 5’-CGCGCCATCCAGGTGTATTT-3’ R: 5’-CTGGTACAGCGTTCCCATGT-3’	123 bp
*hsp70*	FBgn0013279	F: 5’-CGGAGTCTCCATTCAGGTGT-3’ R: 5’-GCTGACGTTCAGGATTCCA-3’	160 bp
*rpl32*	FBgn0002626	F: 5’-GACCATCCGCCCAGCATAC-3’ R: 5’-CGGCGACGCACTCTGTT-3’	138 bp

F: forward, R: reverse.

## Data Availability

Data are contained within the article.
